# INVESTIGATING GROUP B STREPTOCOCCUS (GBS) GENETIC FACTORS ASSOCIATED WITH VERTICAL TRANSMISSION AND INVASIVE NEONATAL DISEASE IN NIGERIA

**DOI:** 10.51894/001c.123111

**Published:** 2024-08-30

**Authors:** Jade E. Le Bris

**Affiliations:** 1 College of Osteopathic Medicine Michigan State University

## 90

### INTRODUCTION

Group B Streptococcus (GBS) is an opportunistic pathogen that asymptomatically colonizes the genitourinary tracts of pregnant patients and can be transmitted to their neonate before or during childbirth. Transmission can result in severe early- or late-onset neonatal disease, preterm births, or stillbirths. Intrapartum antibiotic prophylaxis (IAP) is commonly used to prevent neonatal infection, however, it is not implemented in Nigeria where vertical transmission is common (48.5%) and GBS-associated neonatal morbidities and mortalities are high (early-onset disease incidence of 2.0 cases per 1000 live births).

### OBJECTIVE

Little is known about mutations that may arise in GBS following vertical transmission or whether certain mutations are associated with enhanced transmission or invasive disease. This study utilizes a collection of paired GBS isolates from Nigerian parents and their neonates to identify mutations and genes that are associated with transmission and invasive neonatal disease.

### METHODS

Whole-genome sequencing was performed on 76 GBS isolates recovered from the rectal-vaginal tracts of 38 pregnant patients not treated with IAP and the external auditory meatus of their newborns (n = 38). Raw reads were trimmed, assembled, and checked for quality. Antibiotic resistance and virulence genes were extracted from high-quality reads.

### RESULTS

Among the 76 isolates, only eleven assemblies passed quality control checking, including one mother-newborn pair. The eleven assembled genomes from six mothers and five newborns represented five sequence types and four serotypes and contained 42 different virulence genes; 26 were found in all eleven genomes and included genes for host cell attachment and invasion. Eleven unique antibiotic resistance genes were identified, providing resistance against macrolide, tetracycline, and phenicol antibiotics. Additionally, 45% (n = 5 isolates) were multi-drug resistant with resistance to three or more distinct antibiotic classes.

### CONCLUSION

Although only 11 genomes could be examined due to poor sequencing quality, the data highlight the genetic diversity of this subset that contains a large number of resistance and virulence genes. We expect to obtain similar results after resequencing and completing this analysis for the remaining 65 genomes. Future work including point mutation analysis will further define genomic signatures associated with enhanced mother-newborn transmission and severe neonatal infections.

